# Breast cancer cell lines carry cell line-specific genomic alterations that are distinct from aberrations in breast cancer tissues: Comparison of the CGH profiles between cancer cell lines and primary cancer tissues

**DOI:** 10.1186/1471-2407-10-15

**Published:** 2010-01-14

**Authors:** Katumi Tsuji, Shigeto Kawauchi, Soichiro Saito, Tomoko Furuya, Kenzo Ikemoto, Motonao Nakao, Shigeru Yamamoto, Masaaki Oka, Takashi Hirano, Kohsuke Sasaki

**Affiliations:** 1 Department of Pathology, Yamaguchi University Graduate School of Medicine, Ube 755-8505, Japan; 2 Applied Gene Technology Research Group, Research Institute for Cell Engineering, National Institute of Advanced Industrial Science and Technology, Tsukuba-shi 305-8566, Japan; 3 Department of Surgery, Yamaguchi University Graduate School of Medicine, Ube 755-8505, Japan

## Abstract

**Background:**

Cell lines are commonly used in various kinds of biomedical research in the world. However, it remains uncertain whether genomic alterations existing in primary tumor tissues are represented in cell lines and whether cell lines carry cell line-specific genomic alterations. This study was performed to answer these questions.

**Methods:**

Array-based comparative genomic hybridization (CGH) was employed with 4030 bacterial artificial chromosomes (BACs) that cover the genome at 1.0 megabase resolution to analyze DNA copy number aberrations (DCNAs) in 35 primary breast tumors and 24 breast cancer cell lines. DCNAs were compared between these two groups. A tissue microdissection technique was applied to primary tumor tissues to reduce the contamination of samples by normal tissue components.

**Results:**

The average number of BAC clones with DCNAs was 1832 (45.3% of spotted clones) and 971 (24.9%) for cell lines and primary tumor tissues, respectively. Gains of 1q and 8q and losses of 8p, 11q, 16q and 17p were detected in >50% of primary cancer tissues. These aberrations were also frequently detected in cell lines. In addition to these alterations, the cell lines showed recurrent genomic alterations including gains of 5p14-15, 20q11 and 20q13 and losses of 4p13-p16, 18q12, 18q21, Xq21.1 and Xq26-q28 that were barely detected in tumor tissue specimens. These are considered to be cell line-specific DCNAs. The frequency of the HER2 amplification was high in both cell lines and tumor tissues, but it was statistically different between cell lines and primary tumors (P = 0.012); 41.3 ± 29.9% for the cell lines and 15.9 ± 18.6% for the tissue specimens.

**Conclusions:**

Established cell lines carry cell lines-specific DCNAs together with recurrent aberrations detected in primary tumor tissues. It must therefore be emphasized that cell lines do not always represent the genotypes of parental tumor tissues.

## Background

Cancer cell lines are routinely used for various kinds of biomedical research under the assumption that cell lines reflect the genotypic and phenotypic characteristics of primary tumor tissues. However, such cell lines do not always faithfully represent genomic alterations and gene expression observed in tumor tissue specimens [1-4], and therefore the use of cell lines may lead to erroneous conclusions in some instances. In order to avoid erroneous conclusions in experiments using the cell lines, first of all, it is important to clarify the extent of similarities and differences in genomic aberrations between cancer cell lines and primary cancer tissues.

According to the commonly accepted model for cancer development, somatic mutations accumulate in a cell in the process of tumorigenesis. In clinically overt cancers, not only a large number of genomic aberrations are detected but also genomic instability successively yields genomic alterations in a cancer cell. This theory explains why the number of genomic aberrations is greater in advanced cancers than in early cancers [5-8]. The established cell lines also undergo genomic changes with multiple passages in culture [9-11]. Some of the genomic alterations detected in the cell lines are considered as a result of selective pressure to adapt to the culture conditions, while others may be just incidental [12,13]. This theory raises an additional question in regard to whether there are genomic aberrations specific for cell lines, or *in vitro*-specific genomic aberrations. In this context, it is crucial to distinguish genomic aberrations in tumor tissues from the secondary changes with cultivation. The differentiation between these aberrations is practically difficult, because available data on difference in the genomic changes between cell lines and tumor tissue specimens are very limited at present [13,14]. The comparison of genomic profiles obtained from cell lines with those from primary tumor tissues is one of the best ways to determine the difference in genomic aberrations between cell lines and primary tumor tissues and to identify recurrent celll lines-specific genomic aberrations.

This study examined the DNA copy number aberrations (DCNAs) of 24 breast cancer cell lines and 35 primary breast cancer tissues using array-based comparative genomic hybridization (aCGH). The present paper showed that the breast cancer cell lines preserved genomic alterations detected in primary cancer tissue specimens and that the cell lines concurrently carried secondary genomic alterations. Some of the secondary genomic alterations were recurrent and cell line-specific.

## Methods

### Cell lines

This study used 24 cell lines established from human breast cancer as follows: AU565, HCC2218, T-47D, HCC1954, MDAMB361, UACC812, UACC893, BT474, SKBR3, HCC38, HCC1008, ZR-75-30, HCC1937, MDAMB468, HCC1428, ZR-75-1, MCF7, MDAMB231, MDAMB435S (possibly derived from melanoma), BT483, HCC1806, Hs578T, MDAMB175VII and MDAMB415. These cell lines were purchased from American type Culture Collection (Manassas, VA). The original histology of these cell lines was as follows: 6 adenocarcinomas, 10 invasive ductal carcinomas, and 8 unknown tumors [15].

### Tumor specimen

Thirty-five primary breast cancers that were histologically classified as invasive duct carcinoma were used. All tumors were considered to be sporadic. The average age of patients was 57.6 years, ranging from 31 to 75 years old. In this series, the expression of estrogen and progesterone receptors was positive for 27 (77.1% of tumors) and 19 (54.3%) tumors, respectively. The Institutional Review Board for Human Use Yamaguchi University Graduate School of Medicine approved the study protocol and informed consent for this study was obtained from all patients. A tissue microdissection technique was used to reduce the contamination of samples by normal tissue components for array CGH analyses, as previously described [16]. As a result, the normal cell contamination of samples was usually reduced to less than 10%.

### Genomic DNA

High-molecular-weight DNA was extracted from each tumor specimen with a DNA extraction kit (SepaGene, Sankojyunyaku Co., Tokyo, Japan) according to the manufacturer's instructions as previously described [17-21].

### Array-based CGH

The BAC DNA array used in this study consists of 4030 human bacterial artificial chromosome (BAC) clones, including 356 cancer-related genes, which are spaced approximately 1.0 Mb across the whole genome (MacroGen, Inc., Seoul, Korea). BAC chip information including data of end-sequenced BAC clones is available on the following websites: http://www.macrogen.co.kr/eng/biochip/karyo_summary.jsp. The experiments were performed as previously described [19-22]. Briefly, tumor DNA and gender-matched reference DNA (Promega, Madison, WI) were labeled with Cy5 and Cy3-dCTP (PerkinElmer Life Science, Inc.), respectively, with a random primer labeling kit (BioPrime^® ^DNA Labeling System, Invitrogen™). For hybridization, labeled DNA was mixed with Cot-1 DNA (50 mg, Gibco BRL, Gaithersburg, MD) and ethanol precipitated. The precipitated DNA was dissolved in 40 μl of hybridization mix. The probe mixture was denatured at 75°C for 5 min and incubated at 37°C for 60 min for blocking of repetitive sequences. Arrays were prehybridized with salmon sperm DNA to reduce nonspecific background staining. The probe mixture was applied to the array. The arrays were placed in a moist chamber at 37°C for 72 hr for hybridization. The array slides were washed 2 times in 2× standard saline citrate (2 × SSC), 50% formamide, pH 7.0, at 45°C. The array slides were washed in phosphate buffer with 0.1% NP-40, pH 8.0, once in 2 × SCC at room temperature.

### Imaging and analysis

After hybridization, the slides were scanned on a GenePix 4000A scanner (Axon Instruments, Union City, CA) and the 16-bit TIFF images captured using GenePix Pro 5.0 software. Fluorescence images were analyzed with the MAC Viewer™ software program (Macrogen Inc.) optimized for analysis of the array as previously reported [20-22]. Fluorescence spots were defined with the automatic grid feature and adjusted manually. Then the ratio of the red/green channel of each clone was calculated and converted to a log_2 _ratio. The clones with log_2 _ratios that exceed least than ± 0.25 were considered gain and loss of the copy number. We defined the log_2 _ratio >1.0 as amplifications. A part of the cell line CGH data has been previously reported [23].

### Statistical analysis

The clone-by-clone comparison of the copy number was made between the cell lines and tumor tissue specimens. The differences in the prevalence of common gains and losses between cell lines and tumor tissues were determined with the chi-square test. Differences in the total number of changes and frequency were tested by Student *t*-test. In this study, the Bonferroni correction was made to adjust the p-value. A difference was considered to be significant when the P-value was less than 1.25 × 10^-5 ^(0.05/4030).

## Results

**Figure 1:**
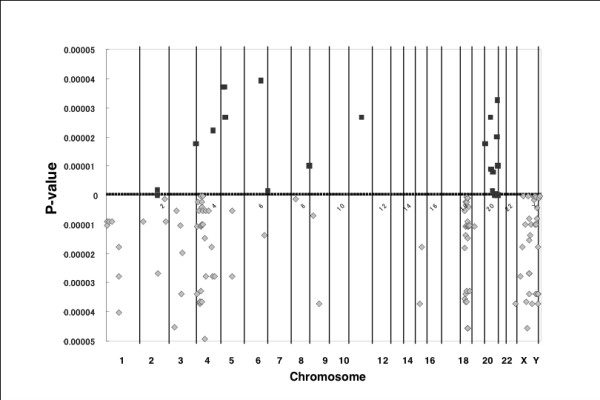
**The frequency of DCNAs detected by array-based CGH in the breast cancer cell lines (n = 24) (a) and the primary breast cancer tissues (n = 35) (b)**. Note the apparent similarity of the DCNA frequency pattern between two groups, gains of 1q, 8q, 17q and chromosome 20 and losses of 8p, 11q, 16q and 17p are frequent in both groups. However, the frequency of DCNA at each chromosomal region is different between these two groups, and other recurrent DCNAs are detected in the cell lines. Gains of 5p and 20q and losses of 4p, 18q and Xq are highly frequent in the cell lines in comparison to the tumor tissues. Green lines denote the frequency of DNA copy number gain in each BAC clone and red lines denote the frequency of DNA copy number loss in each BAC clone. Ordinate; frequency of DCNA for each BAC clone on the array, abscissa; chromosome number.

**Figure 2:**
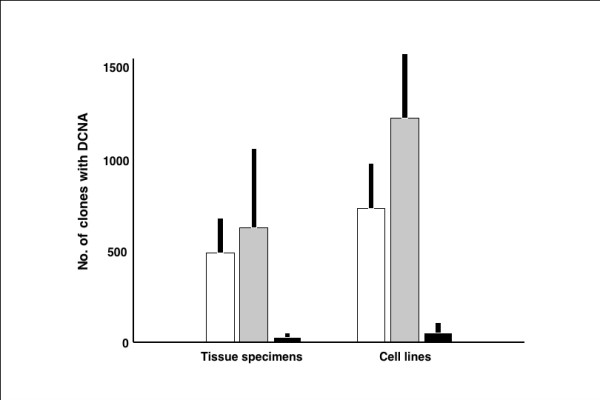
**The average number of DNA copy number gain, loss and amplification for cell lines (n = 24) and the tumor tissues (n = 35)**. The average number of clones with copy number gain is and 651.7 ± 148.8 and 424.2 ± 215.9 in the cell lines and tumor tissues, respectively. The average number of clones with copy number loss is 1180.7 ± 433.8 and 548.0 ± 324.7 in the cell lines and tumor tissues, respectively. The average number of amplification clones is 41.3 ± 29.9 and 15.9 ± 18.6 in the tumor tissues and the cell lines, respectively. The difference is statistically significant (P = 0.012). White columns; the average number of DNA copy number gain. Gray columns; the average number of DNA copy number loss. Black columns; the average number of amplifications. Black bars; standard deviations. Ordinate; frequency of DCNA for each BAC clone, abscissa; samples.

CGH profiles were considerably different between the cell lines and tumor tissue specimens (Figure 1), and the array data reported in this manuscript are available on the following websites: http://cibex.nig.ac.jp/cibex2/ExperimentMiame.do?queryExperimentalDesignAccession=CBX105. The number of DCNAs was more in the cell lines than in the tissue specimens as a general trend. The average number of copy number gains and losses were 651.7 ± 148.8 (standard deviation) and 1180.7 ± 433.8, respectively in cell lines, while they were 424.2 ± 215.9 and 548.0 ± 324.7 in the tumor tissue specimens (Figure 2). However, no statistical difference was found between these two groups. The average number of clones with DNA amplification was statistically different between the cell lines and the tissue specimens (P = 0.012), 41.3 ± 29.9 for the cell lines and 15.9 ± 18.6 for the tissue specimens (Figure 2).

DCNAs were frequently detected on the all chromosomes in the cell lines, but inter-regional differences in the frequency were apparent. DCNAs detected In >50% of the cell lines were as follows: clones with copy number gains and losses were detected at 1q, 5p, 8q and 20q and at 1p, 3p, 4p, 6p, 8p, 9p, 10q, 11p, 13q, 15q, 17p, 18q and X, respectively (Figure 1a). DCNAs detected in >50% of the tissue specimens were as follows: gains of 1q and 8q, and losses of 8p 11q, 16q and 17p were detected (Figure 1b). There were DCNAs shared by the cell lines and tumor tissue specimens and recurrent DCNAs in the tissue specimens were generally frequent in the cell lines. The copy number gains of 1q and 8q were frequent in both the cell lines and tissue specimens (Table 1). In particular, the gain of 1q44 was detected in 13 (54.3%) of 24 cell lines and in 19 (54.3%) of 35 tissue specimens. The gain of 8q22.1 was detected in 15 (62.5%) of the cell lines and in 22 (62.9%) of the tissue specimens. The copy number losses of 11q and 17p were frequent in both the cell lines and the tissue specimens. Notable copy number losses of 17p11.2 and 11q23.2 were frequent; 17p11.2 loss was detected in 13 (54.2%) of the cell lines and 19 (54.3%) of the tissue specimens and 11q23.2 loss was detected in 15 (62.5%) of the cell lines and 22 (62.9%) of the tissue specimens (Table 1).

**Table 1:** Clones with significant difference in frequency of copy number changes between cell lines and tumor tissue specimens

A: Clones with significant difference in frequency of copy number gain between cell lines and tumor tissue specimens				
**Chromosomal region**	**Candidate genes**	**Frequency (%)**		**P-value**
			
		**Cell lines**	**Tissue**	

20q13.33	ZGPAT, BTBD4,	18/24	1/35	5.68551E-09
20q13.13	COX6CP2	18/24	1/33	1.26370E-08
2q22.3		16/24	0/34	2.20899E-08
20q13.31	TFAP2C, PTMAP6	20/24	6/35	4.89015E-07
7p22.3	FLJ20397, UNC84A,	14/24	1/35	1.52990E-06
20q13.12	MYBL2	14/24	1/35	1.52990E-06
20q13.12	ADA, WISP2	14/24	2/35	7.97121E-06
20q11.21	DNMT3B, MAPRE1	11/24	0/35	8.97575E-06
20q11.21-20q11.22	SNTA1	11/24	0/35	8.97575E-06
8q24.3	HSF1, DGAT1, SCRT1	17/24	5/35	1.02265E-05
20q13.33	TPD52L2, DNAJC5	17/24	5/35	1.02265E-05
3q29	LRCH3, IQCG,	12/24	1/35	1.77298E-05
20p13	DEFB32, TRIB3	12/24	1/35	1.77298E-05
20q13.33	ARFGAP1, CHRNA4	19/24	8/35	2.00152E-05
5p14.1		13/24	2/35	2.68521E-05
11q13.3-11q13.4	PPFIA1, CTTN, SHANK2	13/24	2/35	2.68521E-05
20q11.21	BCL2L1, TPX2, MYLK2	13/24	2/35	2.68521E-05
20q13.33	KCNQ2, EEF1A2, PTK6,	17/24	6/35	3.26970E-05
5p15.31		15/24	4/35	3.72069E-05
5p15.2	MARCH6	15/24	4/35	3.72069E-05
5p15.1	BASP1, FTHL10	15/24	4/35	3.72069E-05
6q22.31		16/24	4/31	3.94622E-05
8q24.21	MYC, PVT1,	22/24	14/35	6.40836E-05
7q11.23	POR, TMPIT, DUSP24	9/24	0/35	8.30527E-05
14q22.2-14q22.3	GALIG, LGALS3 DLG7	9/24	0/35	8.30527E-05
19q13.43	ZNF544, ZNF8, HKR2	9/24	0/35	8.30527E-05
20p11.23	ZNF339, RPL15P1	9/24	0/35	8.30527E-05
3q29	TMEM44, FLJ11301	12/24	2/35	8.56953E-05
10p15.3	GTPBP4, IDI2, IDI1	12/24	2/35	8.56953E-05
20p13	CSNK2A1	12/24	2/35	8.56953E-05
20q13.2	ZNF217	17/24	7/35	9.43882E-05

**B: Clones with siginificant difference in frequecy of copy number loss between cell lines and tumor tissue specimens**				

**Chromosomal region**	**Candidate genes**	**Frrequency (%)**		**P-value**
			
		**Cell lines**	**Tissue**	

Xp11.3	UTX	21/24	1/35	3.98705E-11
Xq27.1	-	16/24	0/35	1.52947E-08
Xq21.1	-	18/24	2/35	3.33574E-08
4p15.1	-	16/24	1/35	1.05951E-07
4p13	-	14/24	0/34	3.16890E-07
18q12.3	RIT2	16/23	2/34	3.88478E-07
Xq26.2	OR2AF1	15/24	1/35	4.14685E-07
Xq27.3	HCP44	15/24	1/35	4.14685E-07
18q21.1	MAPK4	18/24	4/35	7.03535E-07
Xq28	F8, VBP1, RAB39B, CLIC2 PHF10P1	13/24	0/35	8.17519E-07
18q21.1	KIAA0427	17/24	3/34	9.90978E-07
8p12	WRN	18/24	4/34	1.01721E-06
2q34	SPAG16	13/24	0/34	1.10437E-06
Xq26.2	GPC3	14/24	1/35	1.52990E-06
4p16.3	FLJ35816	15/24	2/35	2.23384E-06
4p15.1	-	15/24	2/35	2.23384E-06
18q12.2	-	15/24	2/35	2.23384E-06
Xq28	SLC14A2, SLC14A1	15/24	2/35	2.23384E-06
18q21.1	KIAA0427	17/24	3/34	9.90978E-07
8p12	WRN	18/24	4/34	1.01721E-06
2q34	SPAG16	13/24	0/34	1.10437E-06
Xq26.2	GPC3	14/24	1/35	1.52990E-06
4p16.3	FLJ35816	15/24	2/35	2.23384E-06
4p15.1	-	15/24	2/35	2.23384E-06
18q12.2	-	15/24	2/35	2.23384E-06
18q12.3	SLC14A2, SLC14A1	15/24	2/35	2.23384E-06
4p15.1	-	17/24	4/34	4.02576E-06
18q21.32	-	17/24	4/34	4.02576E-06
Xq28	CSAG2, MAGEA2B MAGEA12, CSAG1	14/23	2/35	4.27750E-06
4q13.1	EPHA5	8/16	0/35	5.21729E-06
3p22.1	NKTR, ZNF651, KBTBD5	13/24	1/35	5.34274E-06
4p16.3	HD,	13/24	1/35	5.34274E-06
4p13	-	13/24	1/35	5.34274E-06
4q22.1	-	13/24	1/35	5.34274E-06
5q14.3	-	13/24	1/35	5.34274E-06
18q12.1	-	13/24	1/35	5.34274E-06

**Figure 3:**
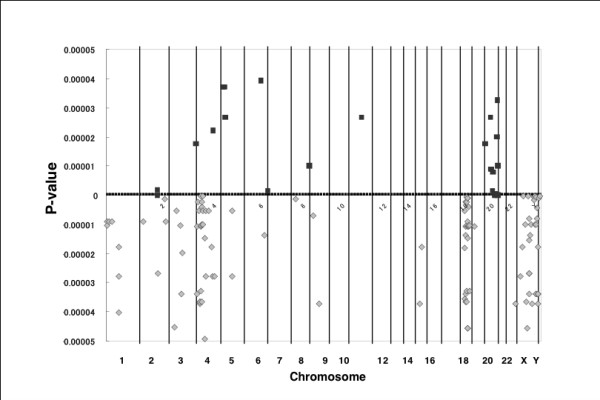
**P-values and chromosomal regions with significant difference in the frequency of DNA copy number gains (black square in upper part) and losses (gray diamond in lower part)**. Clones with cell line-specific copy number gains are densely found at 20q11 and 20q13, whereas clones with cell line-specific losses are detected at 4p13-14, 18q12, 18q21, Xq21 and Xq26-28. This figure shows clones with highly significant difference (p < 0.00005) in the frequency between the cell lines and the tumor tissues. Ordinate; statistic P-values, abscissa; chromosome number.

The clone-by-clone comparison of the DCNAs between the cell lines and the tumor tissues provided detailed information concerning the difference in DCNAs between two different sample groups. Gains of 5p14-p15, 20q11 and 20q13 and losses of 4p13-p16, 18q12, 18q21, Xq21.1 and Xq26-q28 were detected almost exclusively in the cell lines (Figure 3). For instance, the copy number gain of clones located on 20q13.33 and 20q13.13 were detected in as many as 75% of the cell lines, but it was a rare event (around 3% of tumors) in the primary tumor tissues (P = 5.68 × 10^-9 ^and P = 1.23 × 10^-8^, respectively)(Table 2). The frequency of the Xq27.1 loss was detected in 16 (66.7%) of the cell lines, but not detected in the tissue specimens (P = 1.53 × 10^-8^) (Table 2).

**Table 2:** Clones with recurrent copy number changes shared by cell lines and tissue specimens

A: Clones with recurrent copy number gains shared by cell lines and tissue specimens				
**Chromosomal region**	**Genes**	**Frequency**		**P-value**
			
		**Cell lines**	**Tissue**	

1q44	FLJ10157	13/24	19/35	0.992806112
8q22.1	TSPYL5	15/24	22/35	0.977767962
8q21.3	NBS1, DECR1	14/24	20/35	0.927569885
8q23.1	MGC35555	14/24	20/35	0.927569885
1q21.1	-	12/24	18/35	0.914136773
1q21.2-1q21.3	PIP5K1A, PSMD4, KIAA1441	12/24	18/35	0.914136773
1q23.1	SH2D2A, INSRR, NTRK1	12/24	18/35	0.914136773
1q32.1	MDM4	12/24	18/35	0.914136773
1q42.11	CAPN2, TP53BP2	12/24	17/35	0.914136773
1q44	FLJ32001, CGI-49	12/24	17/35	0.914136773
1q44	OR1C1, OR9H1P, OR11L1	12/24	18/35	0.914136773
7p14.1	TRGJP2, TRGC1, TRGJ1	12/24	18/35	0.914136773
7p14.1	TRGJP1, TRGV11, TRGVB	12/24	17/35	0.914136773
8q24.22	-	12/24	17/35	0.914136773
17q25.3	TBCD	12/24	17/35	0.914136773
1q25.1	TNN, KIAA0040	14/24	21/35	0.898133861
8q21.3	NBS1	14/24	18/35	0.898133861
8q22.2	KCNS2, STK3	14/24	18/35	0.898133861

**B; Clones with recurrent copy number losses shared by cell lines and tissue specimens**				

**Chromosomal region**	**Genes**	**Frequency (%)**		**P-value**
			
		**Cell lines**	**Tissue**	

17p11.2	DRG2, MYO15A, LLGL1, FLII	13/24	19/35	0.992806112
17p12	LOC388338, HS3ST3B1	15/24	22/35	0.977767962
11q23.2	ZBTB16	12/24	18/35	0.914136773
11q25	SPAS1	'12/24	18/35	0.914136773
17p13.3	NXN	'12/24	17/35	0.914136773
17p13.1	ASGR1, DLG4, ACADVL	14/24	21/35	0.898133861
17p13.1	MYH3, SCO1, MDS006	14/24	19/34	0.852726956

The frequency of amplification was higher in the cell lines than in the tissue specimens (Table 3). DNA amplification was frequent at a wide chromosomal region 17q12-q21 in both the cell lines and tissue specimens, though other BAC clones with frequent DNA amplification were considerably different between these two sample groups (Table 3). The amplification of the chromosomal region 17q12 including ERBB2/HER2 was detected in 10 (41.7%) of 24 cell lines and 6 (17.1%) of 35 tissue specimens (P = 0.037).

**Table 3:** Clones with frequent DNA amplification in breast cancer cell lines and primary tumor tissues

Chromosomal region	Candidate genes	No, of tumors	Frequency (%)
**Cell lines (n = 24)**			
17q12	NEUROD2, PPP1R1B, STARD3, TCAP, PNMT, PERLD1	10	41.7
17q12	PERLD1, **ERBB2**, GRB7, ZNFN1A3	10	41.7
17q21.1	ZNFN1A3, ZPBP2, GSDML, ORMDL3, GSDM1, PSMD3	8	33.3
8q24.13	ZHX2	7	29.2
5p15.33	TPPP, LOC442127, ZDHHC11	6	25.0
8q24.12	SAMD12	6	25.0
8q24.12	MRPL13, MTBP, SNTB1	6	20.5
8q24.22	TG,	6	25.0
20q13.2	BCAS1, CYP24A1	6	25.0
20q13.2	BCAS1	6	25.0
20q13.2	DOK5	6	25.0
20q13.2	DOK5	6	25.0
20q13.32	PCK1, ZBP1, TMEPAI,	6	25.0
**Tumor tissues (n = 35)**			
17q12	NEUROD2, PPP1R1B, STARD3, TCAP, PNMT, PERLD1	7	20.0
17q12	PERLD1, **ERBB2**, GRB7, ZNFN1A3	8	17.1
17q21.1	ZNFN1A3, ZPBP2, GSDML, ORMDL3, GSDM1, PSMD3	6	17.7
8p12	-	5	14.3
8q21.11	PI15	5	14.3
8q21.11	ZFHX4	5	14.3
8q24.21	DDEF1	5	14.3
17q23.3	TEX2	5	14.3
8p12	WHSC1L1, LETM2, FGFR1	4	11.4
8p12-8p11.23	LETM2, FGFR1	4	11.4
8p11.23	TACC1, PLEKHA2	4	11.4
8q21.11	-	4	11.4
8q21.12	IL7	4	11.4
8q21.3	RUNX1T1	4	11.4
8q22.1	-	4	11.4
8q22.3	-	4	11.4
8q23.3	-	4	11.4
8q24.11	-	4	11.4
8q24.13	-	4	11.4
17q23.3	CSH1, CSHL1, GH1, CD79B, SCN4A	4	11.4

-: The relevant clone harbors no genes identified.

The number of clones with DNA amplification that is detected in more than 10% of samples is 101 clones in the cell lines, while it is only 20 clones in tumor tissues.

## Discussion

Making a comparison of the CGH profiles between the established cell lines and their parental tumor tissue specimens is practically impossible, because the source tissue specimens are no longer obtainable. Therefore, the comparison of the genomic profiles obtained from cell lines with those from primary tumor tissues is one of the best ways to determine the difference in genomic aberrations between cell lines and primary tumor tissues and to identify recurrent cell lines-specific genomic aberrations.

The array-based CGH revealed a large number of DCNAs including recurrent ones in both breast cancer cell lines and primary breast cancer tissues. There was a tendency that the average number of DCNAs was greater in cell lines than in primary breast cancer tissue specimens, 1832.4 (45.5% of spots) and 972.2 clones (24.1%) for a cell line and tumor tissue, respectively. This result is consistent with the data reported by Naylor and colleagues [24]. The comparison of CGH profiles between cell lines and tumor tissues revealed gains of 1q and 8q and losses of 8p, 11q, 16q and 17p as recurrent DCNAs shared by two groups. Although there are some variations in the CGH patterns of breast cancers between studies, copy number gains of 1q, 8q, 11q, 17q and 20q and losses of 6q, 8p, 9p, 13q, 16q and 17p were previously reported as recurrent aberrations in breast cancers [14,15,24-27]. Gains of 1q44, 1q21 and 8q21-q23 and a loss of 17p11-p13 were detected in both of the cell lines and the tumor tissues at high rates (>50% of both samples). The present observations support the hypothesis that the cell lines basically preserve the genomic alterations that have occurred in primary tumor tissues [13,24]. These recurrent DCNAs detected in both cell lines and tumor tissues are though to be closely relevant to the development and progression of breast cancer. The clone-by-clone comparison of DNA copy numbers between cell lines and tumor tissues allowed detection of recurrent DCNAs exclusively in breast cancer cell lines as well as recurrent DCNAs shared by two groups. Gains of 5p14-15, 20q11 and 20q13 and losses of 4p13-p16, 18q12, 18q21, Xq21.1 and Xq26-q28 were detected almost exclusively in the cell lines. Although the resolution of the BAC array used in this study is low in comparison to the tiling arrays, this study revealed a distinct difference in the patterns of the copy number aberrations between primary tumor tissues and cell lines. When data of cell lines are compared between the present CGH platform and others including tiling arrays, the chromosomal regions identified as copy number aberrations in this study are compatible with those provided by tiling arrays [27,28]. Indeed, gains of 8q and 20q were frequently detected by the 4K array slides as well as by tiling arrays. The present data provided by the 4K array platform are thus considered to be reliable.

It is particularly worth noting that some of recurrent DCNAs that are frequently detected in cell lines are hardly detected in primary cancer tissues. These DCNAs correspond to cell line-specific or *in vitro-*specific alterations [13,24,29]. Interestingly, these recurrent DCNAs identified in breast cancer cell lines were also detected in cell lines of other types, such as gastric cancer [30], lung cancer [31], colon cancer [32] and pancreatic cancer cell lines [33]. Established cell lines possibly carry the cell line-specific DCNAs regardless of the parental tumor types. DCNA profiles primarily depend on organs and tissues from which cancer develops [13,34,35]. Breast epithelial cells transformed *in vitro *show genomic alterations similar to those of cell lines [36]. Therefore, taking these observations into consideration, it is legitimate to consider that *in vitro *environments endow cells with genomic aberrations of which cell line-specific DCNAs are critical for cells to survive and proliferate *in vitro*. Indeed, it is known that the pattern of CGH profiles change in varying degree with the cell passage numbers [9].

The difference in the CGH profiles found between the cell lines and tumor tissues is not attributed to just variations in genomic alterations between parental histological types, because DCNAs detected exclusively in cell lines are not explained by the difference in histologic types [37-39]. Accordingly, this also strengthens the view that recurrent DCNAs detected exclusively in cell lines can be called cell line-specific aberrations.

The average number of amplified clones was more than double in the cell lines than the tumor tissues (41.3 vs. 15.9). In particular, the frequency of amplification for clones on 17q12-q21, encompassing many genes including ERBB2/HER2 that is the most frequent amplified gene in breast cancers [40,41], was higher in the cell lines than in tissue specimens. The amplification frequency of the BAC clone harboring ERBB2/HER2 was in 41.7% of the cell lines and 17.1% of the tumor tissues. The amplification of ERBB2/HER2 is usually detected in no more than 20% of breast cancer patient [42]. Therefore, it can be said that additional DNA amplifications occur not only in the chromosomal region but also in other regions in the cell lines.

## Conclusions

The cell lines carry the cell lines-specific DCNAs together with *in vivo *alterations. Cell line-specific DCNAs were as follows: gains of 5p14-15, 20q11 and 20q13 and losses of 4p13-p16, 18q12, 18q21, Xq21.1 and Xq26-q28. When cell lines are used as an alternative to primary tumor tissues, it is therefore important to keep in mind that cell lines do not always represent genotypes of parental tumor tissue specimens.

## Abbreviations

CGH: comparative genomic hybridization; DCNAs: DNA copy number aberrations; BAC: bacterial artificial chromosome

## Competing interests

The authors declare that they have no competing interests.

## Authors' contributions

KT, SK and TM carried out array CGH of tumor tissues, SS and TH were invovled in array CGH of cell lines, SY and MO gathered tumor tissue specimens and clinico-pathological data, MN analyzed array CGH data. KT and SK organized the array CGH data, and KT drafted the manuscript. KS conceived of this study, and participated in its design and coordination. All authors read and approved the final manuscript.

## Pre-publication history

The pre-publication history for this paper can be accessed here:

http://www.biomedcentral.com/1471-2407/10/15/prepub
